# Effect of school feeding program on body mass index of primary school adolescents in Addis Ababa, Ethiopia: A prospective cohort study

**DOI:** 10.3389/fnut.2022.1026436

**Published:** 2023-01-09

**Authors:** Bekri Mohammed, Tefera Belachew, Shemsu Kedir, Kalkidan Hassen Abate

**Affiliations:** ^1^Department of Human Nutrition, Institute of Public Health, College of Medicine and Health Sciences, University of Gondar, Gondar, Ethiopia; ^2^Department of Nutrition and Dietetics, Food and Nutrition Research Institute, Jimma University, Jimma, Ethiopia; ^3^Department of Public Health, College of Medicine and Health Science, Werabe University, Werabe, Ethiopia

**Keywords:** school feeding program, body mass index, adolescents, BAZ, school

## Abstract

**Background:**

Governments and developmental organizations are encouraged to devote adequate resources to the establishment of free school meals to low-income children in developing countries. In Ethiopia, the school feeding program (SFP) is implemented in a few regions including the capital, Addis Ababa. However, the nutritional benefit of the program was not monitored and reported thus far. In this study, we evaluated the effect of the SFP on the body mass index (BMI) of primary school adolescents in Addis Ababa, Central Ethiopia.

**Method:**

A prospective cohort study was employed to collect data from 644 SFP-beneficiary adolescents (*n* = 322) and non-SFP (*n* = 322). Nutritional outcomes were measured following 6 months of follow-up. WHO Anthroplus were used to convert anthropometric data into body-mass-index-for-age Z scores. The independent effect of school feeding is analyzed through a multivariable linear regression model.

**Result:**

In linear regression, unadjusted model (Model 1), compared with the non-school-fed adolescents, the mean difference in difference of BAZ-score of school-fed adolescent was higher by 0.36 (β 0.36, 95% CI 0.17, 0.55). The beta coefficient remained positive after adjusted for age and sex (Model 2: β 0.35, 95% CI 0.16, 0.54) and sociodemographic variable (Model 3: β 0.35, 95% CI 0.16, 0.54). In the final model, adjusted for model four, lifestyle and health status there was a significant difference in favor of school-fed adolescents on BAZ-score indices (Model 4: β 0.4, 95% CI 0.18–0.62). On the contrary, for a unit increase in family size, the BAZ score will decrease by 0.06 (β 0.06, 95% CI −0.12–−0.01). Similarly, adolescents with a middle tertile wealth index decreased their BAZ score by 0.30 (β 0.30, 95% CI −0.55–−0.05) as compared to the higher tertile wealth index.

**Conclusion:**

School feeding was positively associated with a change in BAZ score whereas family size and middle tertile wealth index were negatively associated. This implies that school feeding can serve as an optimal strategy for addressing the nutrition needs of adolescents.

## Introduction

According to UNICEF, adolescence encompasses children of age group between 10 and 19 years (1). The Global estimate of the adolescent population is 1.2 billion (16 percent of the total population), while the sub-Saharan African region is known to have the greatest proportion (23%). In Ethiopia, about a quarter of the general population is estimated to be adolescents (2, 3). Adolescence is the second rapid growth period and psychosocial development which can determine the upcoming adult health conditions (4). Unmet nutritional requirements sub-optimal dietary diversity and inadequacies are the major threats during this period. Moreover, psychosocial factors play a major role in adolescent health and dietary decisions (5–7).

Stunting or wasting are affecting more than 200 million children in low- and middle-income countries (8). Likewise, as many almost twice suffer from essential micronutrient deficiencies. On the other hand, the number of overweight and obese children is on the rise, bringing a dual burden of malnutrition on the frail health care system (9). In East and Central Africa, nearly one-third of all boys were underweight while the corresponding figure for girls ranged from 15.52 percent in Rwanda to 22 percent in Ethiopia (8). According to the global burden of disease recent estimate, in Ethiopia, protein-energy malnutrition prevalence among adolescents aged 10 to 14 and 15 to 19 was 33 and 38% respectively (10).

Child health, growth, and development are outcomes of direct and indirect linked multi-layered factors (2). The UNICEF global conceptual framework depicts the causes of malnutrition in terms of immediate, underlying, and enabling. The immediate determinants are diets and health, which also influence each other. In the adolescent context, their mental wellbeing also plays a significant influence on their dietary intake and physical activity. Mental health conditions may affect mood and desire to eat, disrupt the ability to digest food or absorb nutrients, and cause a person to neglect their wellbeing and forget to eat (11, 12).

The underlying determinants are the food practices, and services available to children and women in their communities. The last enabling determinants are the financial, political, cultural, social, and environmental conditions (13).

There exist a wide array of interest by several global organizations including WHO, FAO, and UNICEF in addressing the nutrition need of adolescents where schools were used as an optimal setting for interventions (1, 14, 15). Several interventions focused on promoting nutrition education, provision of food, improving quality of diets in schools and beyond, promoting healthy food environments, promoting healthy dietary practices, providing micronutrient supplementation, promotion of exercise, and deworming are known to have been delivered in the school environment (1).

School feeding programs are kind of interventions designed to provide good quality nutrient foods to children and adolescents attending school (16, 17). It is a component of national social protection systems for many countries including those with a low prevalence of undernutrition (17). Most countries had some form of a school feeding program for adolescents like daily snacks or meals (18). However, the design and implementation modality of school feeding varies significantly (19). Novel approaches, such as school farming for school feeding, agroforestry projects relating to school feeding, home grew school feeding have been receiving attention (20, 21). The nutritional benefits of the school feeding program were also anticipated to serve as a tool to improve the educational capabilities of children (22). Alleviating hunger, reducing malnutrition, Improving school attendance and enrollment, improving academic and cognitive achievements, and contributing educational gender equity are reported benefits of school feeding (23–25).

Poverty eradication, nutrition, health, and education, continue to be pillars of sustainable development. holistic school meal programs can make an adequate, sustainable positive impact on these determinants of development through various pathways (26). The National School Health and the Nutrition Strategy and National Nutrition Program of Ethiopia have identified a key nutrition-sensitive intervention by promotion of home-grown SFP to combat malnutrition (27). World Food Program-funded SFP was began in Ethiopia in 1994 (28). Later, in 2016 drought-affected areas were involved to address more than a million children in the program while in 2020 the SFP expand to some urban cities including Addis Ababa (26, 27). Addis Ababa City government provides a school feeding program for about three hundred thousand students aiming at reducing absenteeism and increasing enrollment among school children (29).

Although the school feeding program is initiated in some parts of Ethiopia, the extent of its nutritional benefit among adolescents was not explored enough thus far. In this we hypothesized that school fed children will have improved nutritional status as compared to non-school fed children. Hence, this study was designed to investigate the effect of school feeding on the body-mass-index for z-score of primary school adolescents.

## Methods and materials

### Study area

A prospective cohort study design was conducted among primary school adolescents' in Addis Ababa and Adama, Central Ethiopia. Addis Ababa is the capital city of Ethiopia with great diversity, and homes of almost all ethnicities found in the country. According to the 2018 report of the Addis Ababa City Government Educational Bureau, there are 806 primary schools. In all these schools, there are a total of 504,205 students. Of the total number of schools, 223 (27.66%) are governmental and 583 (72.33%) are private (30). Adama town, central Ethiopia, is found 99 km southeast of Addis Ababa. According to ACAEO statistics records in Adama, there are 11 governmental, 3 public, 6 religious, and 13 local private schools. According to the Adama city administration office, the total number of primary school students are 38,503 (31).

### Populations

Adolescents aged between 10 and 19 in Addis Ababa and Adama enrolled in the primary school for the 2020/2021 academic years who were attending their regular classes were included. Addis Ababa (Exposed group) and Adama (Unexposed group) are two neighboring cities that are found in the central part of Ethiopia. The school feeding program schools (Exposed group) are given to all the government primary schools found in Addis Ababa and it was considered our source population. The Non-school feeding program schools (unexposed group) were taken from the neighboring town, Adama. Regarding the characterization of exposed and unexposed, we have been going through an independent *t*-test to check sociodemographic characteristics and it is insignificant which shows the two groups have similar sociodemographic characteristics.

### Sample size determination

The sample size was calculated using G^*^Power 3.1 program (32) assuming that the primary outcomes would be compared within the two groups using a one-tailed mean difference test. The sample size was calculated with a 95% confidence level, 80% power, medium effect size (d = 0·4), one-to-one allocation ratio between the two groups, and design effect of 2. Further, 5% compensation for possible dropout was added. Ultimately, the sample size of 676 (338 SFP beneficiaries and 338 non-beneficiaries) was determined (33).

### Sampling technique and procedures

A stratified multi-stage sampling procedure was employed to select the study units. In the two cities, there are 223 governmental primary schools in Addis Ababa and 11 in Adama. Sixty-seven and three governmental primary schools were selected by simple random sampling from Addis Ababa and Adama respectively. The total sample size was allocated for each selected primary school proportionally to the number of students within each SFP beneficiaries in Addis Ababa and Non-SFP beneficiaries in Adama primary schools. Finally, study participants were selected by a simple random sampling method from a student list of each section.

### Measurement

#### Anthropometric measurements

Body weight was measured on an electronic digital scale to the nearest 0.1 kg with minimum clothing and baring foot. and height was measured using a measuring instrument (Stadiometer, CE 0123, Germany,) to the nearest 0.1 cm by looking straight ahead with Frankfurt plane horizontal, shoulders relaxed, arms at sides, legs straight and knees together feet flat and with heels almost together. The shoulder blades, buttocks, and heels touch the measurement board. WHO recommend BMI-for-age to assess thinness/wasting in registered school-aged children. Overweight (> + 1SD BMI-for-age z score), obesity (> + 2SD BMI-for-age z score), thinness/ wasting (< −2SD of BMI-for-age z score), stunting (< −2SD of height-for-age (HAZ) z score) were defined according to the WHO references (34, 35).

##### DDS

Food frequency questionnaire containing 28 food items was used to assess dietary diversity score that are commonly consumed in the study area. Wide-ranging interview of key informants from the study area who knew the culture and language were used to develop the list of food items. The food frequency questionnaire was refined based on the result of the pretest on 14 adolescents' responses by observing patterns of week days of common food consumption. Cronbach's alpha result was 0.79 during the pretest. Adolescents were considered as “consumers” of a food item if they had consumed the food item at least once per week. As there is no Ethiopian classification of food groups, the 28 food items of the food frequency questionnaire were grouped into nine groups (36). For instance, an adolescent who consumed one item from each of the food groups at least once during the week would have a maximum DDS of 9, and those who did not consume per week scored 0 for all food groups (37).

##### Physical inactivity

WHO Global Physical Activity Questionnaire (GPAQ) (38) was used to assess the physical activity and sedentary behavior of adolescents. The questionnaire includes items that require participants to indicate the kinds of physical activities that they do as part of their daily activities. The participants were asked on the time spent being physically active in the last 7 days, based on work-related activities, yard work, domestic chores, and activities related to commuting from one place to another, and those undertaken in exercise, sport, or during pastime. The study subject was also asked to think about all the vigorous and moderate activities that they did in the last 7 days. Vigorous physical activities were those that require hard physical effort and make participants breathe much harder than normal. Moderate activities were those that demand moderate physical effort and make them breathe somewhat harder than usual, while low physical activities were those involving walking and being sedentary at least 7 times a week for a minimum of 10 min. In the GPAQ only sessions which lasted 10 min or more were analyzed. All types of physical activities related to occupation, transportation, household chores, and leisure time were also included. Based on the GPAQ scores, their physical activity levels were categorized as follows: Low = METs scores of < 500, Moderate = METs scores of between 500 and 1,499 and Vigorous = METs>1500.

##### Mental health

Mental health status (common mental disorders) were assessed using Self-Reporting Questionnaire (SRQ-20). The SRQ is a 20-item with yes/no answers that measure psychiatric symptomatology which developed by the World Health Organization (39, 40). The SRQ includes both somatic items (e.g., headaches, loss of appetite) and psychological items (e.g., feeling unhappy, nervous, and worthless). It is implemented throughout low-and middle-income countries, including Ethiopia and many other African settings (40–43).

#### Difference-in-differences estimation

We used difference-in-difference (DiD) estimators to compares the changes in outcomes from baseline to end line in SFP and non-SFP:


$$\begin{array}{l} \Delta \text{DID}=E\left[\left({\text{Y}}_{1}^{\text{T}}-{\text{Y}}_{0}^{\text{T}}\right)-\left({\text{Y}}_{1}^{\text{C}}-{\text{Y}}_{0}{\;}^{\text{C}}\right)\right] \end{array}$$

where Y_0_ and Y_1_ denote outcomes at baseline and end line respectively, and T and C denote treatment (SFP) and control (non-SFP).

Formally, our DiD specification is


$$\begin{array}{l} Yivt\;=\text{ β}0+\text{β}1SF+\text{β}2^{*}Round2+\text{β}3^{*}SF^{*}Round2+\text{γ}X\;\\ \;++\sum^{\text{​}}\delta vV+\;\varepsilon ivt \end{array}$$

Where Yivt is the outcome for adolescent i in group v at time t

Round 2 is a time dummy/ follow-up survey

SF is a school feeding/ treatment indicator

SF^*^Round 2 is school feeding^*^time interaction

β3 is DiD estimate

X represents sociodemographic characteristics

V stands for the set of dummy variables for both groups.

εivt is the error term.

#### Analysis

The data were doubly entered into Epi-Data version 3.1 and checked using a side-by-side comparison to check clerical errors. Then the data were exported to SPSS for windows version 24 to check for missing values and outliers, and for further analysis. Principal Component Analysis was performed to generate a household wealth index—a composite index of living standards based on multiple variables including materials used for house building, access to a drinking water source and sanitation facilities, ownership of livestock, house, and agricultural land. A varimax rotation was applied and the Kaiser–Meyer–Olkin measure of sampling adequacy was acceptable (0·64), and Bartlett's test of sphericity was significant. The variables that had communality scores >50% were retained in the analysis. The factor with the highest eigenvalue was taken and then divided into three equal tertiles: poor, middle, and rich. Descriptive analyses were carried out to generate means and proportions and compared them by BAZ score using Chi-square tests.

The Z score values for height-for-age, weight-for-age, and BMI-for-age were calculated using the WHO Anthroplus 2007 reference. As there were baseline differences in some variables between exposed and unexposed groups, the difference in differences was employed in all analyses for comparison of the exposed and unexposed to determine the effectiveness of the school feeding program on BMI Z-score changes. The Difference in Difference (DID) linear regression was performed to assess the effect of the SFP on BMI for age z-score, and the assumptions of the model (normality and homoscedasticity of error terms and linearity of relationship) were assessed using partial plots and found to be satisfied. In all multivariable models, the absence of multi-collinearity was evaluated using the variance inflation factor and found to be within the acceptable range (variance inflation factor < 10).

## Result

Out of the 676 study participants, 663 adolescents completed the questionnaires at baseline and additional 19 students were not included due to dropping out at the end line. We excluded a total of 32 adolescents (An equal number from both exposed and unexposed at random) who were not found in the school. A total of 644 adolescents aged 10–19 years old were enrolled in 78 selected primary schools. Of these, 322 were from the SFP beneficiary group whereas 322 were from the non-SFP beneficiary group and the response rate was 95%. Nearly, 50% of the respondents in non-school feeding and 48% in the school feeding adolescents were female. The mean (SD) age of school feeding and non-school-feeding adolescents were 14.9 (1.86) and 15.04 (1.67) respectively. Similarly, the mean (SD) family size of respondents for school feeding was 5.47 (1.57) and for non-school feeding, adolescents were 5.59(1.77) (Table 1).

**Table 1:** Demographic and socio-economic characteristics of a cohort of primary school adolescents at baseline in Addis Ababa and Adama, Central Ethiopia in 2020/2021.

**Variables**	**SFP**	**Non-SFP**	***P*-value**
Mean (SD) age in year	14.9 (1.86)	15.04 (1.67)	0.33
Mean (SD) family size	5.47 (1.57)	5.59 (1.77)	0.34
**Sex (%)**			
Male	166 (51.6)	160 (49.7)	0.63
Female	156 (48.4)	162 (50.3)	
**Wealth index (%)**			
Low	135 (41.9)	134 (41.6)	0.73
Middle	108 (33.5)	101 (31.4)	
Higher	79 (24.5)	87 (27.0)	
**Household head (%)**			
Male	241 (74.8)	244 (75.8)	0.78
Female	81 (25.2)	78 (24.2)	
**Paternal education (%)**			
Formal education	261 (81.1)	246 (76.4)	0.15
No formal education	61 (18.9)	76 (23.6)	
**Maternal education (%)**			
Formal education	187 (58.1)	183 (56.8)	0.75
No formal education	135 (41.9)	139 (43.2)	
**Paternal occupation (%)**			
Employed	181 (56.2%)	175 (54.3%)	0.63
Unemployed	141 (43.8%)	147 (45.7%)	
**Maternal occupation (%)**			
Employed	117 (36.3%)	111 (34.5%)	0.62
Unemployed	205 (63.7%)	211 (65.5%)	

Chi-square, independent t-test.

The coefficient on the BAZ score is positive and statistically significant. These results indicate that there is a relationship between school feeding program participation and adolescent BAZ-score based on observable and time-invariant characteristics. In Table 2, a comparison of anthropometric indices based on exposure status at baseline and end-line was made and the differences in the differences between the baseline and end-line values were compared. The results showed that there was a significant difference in differences in anthropometric indices. The school feeding program had a high difference of differences in height, weight, and BAZ. The mean difference of differences was higher in the school feeding group by 1.31 cm (p < 0.001) for height, 2.75 kg (p < 0.001) for weight, and 0.36 Z-score (p < 0.001) for BAZ (Table 2).

**Table 2:** Differences in differences between baseline and end-line measurements of anthropometric indices among school feeding and non-school feeding groups of adolescents in Addis Ababa and Adama, Central Ethiopia, 2020/2021.

	**School feeding program**			**Non-school feeding program**			**DID (SFP—Non-SFP) Mean (SE)**	***P***
	**Baseline Mean (SD)**	**End-line** **Mean (SD)**	**Difference** **(EL-BL)1 Mean (SD)**	**Baseline** **Mean (SD)**	**End-line Mean (SD)**	**Difference (EL-BL)1** **Mean (SD)**		
Height	146.5 (7.61)	148.7 (7.62)	2.13 (1.72)	145.9 (8.14)	146.7 (8.18)	0.81 (6.18)	1.31 (0.31)	< 0.001
Weight	39.35 (6.04)	43.57 (7.03)	4.22 (6.72)	39.70 (6.62)	41.17 (7.01)	1.46 (2.60)	2.75 (0.40)	< 0.001
BAZ	−0.62 (1.32)	−0.01 (1.10)	0.60 (1.33)	−0.88 (1.39)	−0.62 (1.44)	0.24 (1.09)	0.36 (0.09)	< 0.001

EL, End-line mean; BL, Baseline mean; DID, Difference in difference (mean difference of SFP—mean difference of Non-SFP); SD, Standard deviation; SE, Standard error.

In linear regression, unadjusted model (Model 1), compared with the non-school-fed adolescents, the mean difference in difference of BAZ-score of school-fed adolescent was higher by 0.36 (β 0.36, 95% CI 0.17, 0.55). The beta coefficient remained positive after adjusted for age and sex (Model 2: β 0.35, 95% CI 0.16, 0.54) and sociodemographic variable (Model 3: β 0.35, 95% CI 0.16, 0.54). In the final model, adjusted for model four, lifestyle and health status there was a significant difference in favor of school-fed adolescents on BAZ-score indices (Model 4: β 0.4, 95% CI 0.18–0.62) (Table 3).

**Table 3:** Multivariable linear regression models predicting mean baseline to end line differences of the differences in BAZ score among school feeding and non-school feeding groups of adolescents in Addis Ababa and Adama, Central Ethiopia, 2020/2021.

**Model**	**β (95% CI) in Z score**	**Covariates**
Model 1	0.36 (0.17, 0.55)^***^	Unadjusted
Model 2	0.35 (0.16, 0.54)^***^	Model 1 adjusted Sex, age
Model 3	0.35 (0.16, 0.54)^***^	Sex, age, family size, wealth index, household head, paternal work, maternal work, paternal education, maternal education
Model 4 (fully adjusted model)	0.40 (0.18, 0.62)^***^	Sex, age, family size, wealth index, household head, paternal work, maternal work, paternal education, maternal education, dietary diversity, work other than being a student at least for 1 h, physical inactivity, time spend on television, computer, and social media and mental health

^*^Significant at *P* < 0.05, ^**^Significant at *P* < 0.01,

^***^Significant at *P* < 0.001, all β-coefficients (95% CI), were from multiple linear regression analysis, and related to the non-exposed groups.

Model 1, Unadjusted; Model 2, biological factors; Model 3, Biological & sociodemographic factors; Model 4, Biological & sociodemographic factors, health and lifestyle factors (fully adjusted).

On the contrary, for a unit increase in family size the BAZ score will decrease by 0.06 (β 0.06, 95% CI −0.12–−0.01). Similarly, adolescents with a middle tertile wealth index were decrease BAZ score by 0.30 (β 0.30, 95% CI −0.55–−0.05) as compared to the higher tertile wealth index.

## Discussion

In this study, the nutritional impact of school feeding was examined by measuring the difference in difference (DID) of the BAZ score of adolescents. In crude comparisons, the findings of the study indicated that the intervention group has a significantly positive impact on the nutritional status of school adolescents. Furthermore, adjusted for all possible covariates, the SF program was found to have a statistically significant positive effect on adolescents' BAZ scores with a coefficient of 0.40. Our finding is in line with a systematic review of randomized controlled trials (RCTs) done in low and middle-income countries that showed a small but significant effect of school meals on weight gain among adolescents (44). Furthermore, a study done in Bangladesh reported that the average BMI of SFP participating students is 0.62 points higher than the average BMI of enrolled children in the control area (45). Cole also suggested the positive impact of the school feeding program on BAZ scores in Lao People's Democratic Republic (46). Similarly, earlier observational studies conducted in Ethiopia (47, 48) in Addis Ababa and Sidamo, reported improvement in BAZ scores following the school feeding program.

On the contrary, there existed other studies which reported the null effect of school feeding programs on Nutritional benefits in Africa (49, 50). One of the key elements which could have a substantial influence on the effectiveness of the SF program was the delivery modality of the SF program. For example, a study from Burkina Faso reported no effect of school meal programs carried out in the form of take-home ration (THR) rather than in school feeding (49). It is plausible to attribute the failure of the THR program to food sharing among household members, compromising its impact on BAZ of school-aged children. In the study conducted in Uganda, the nutritional status did not differ significantly between children who take porridge at mid-morning break and those who do not (50). This study may indicate simple calorie supplements may not be good enough in realizing anthropometric changes. Additionally, the parents of school-fed adolescents reduce the food portion size at home in the sense that they fed at school. Similarly, it may be due to the high level of poverty in the community selected to take SFP. Moreover, the report by the Ghana school feeding program indicated that the SFP faces challenges like the inconsistent release of funds which makes feeding irregular, and loss of meeting the RDA for macro-nutrient (51, 52).

In the midst of these contrasting observations, the available evidence supports the use of school environment can be used to improve the dietary habits of children. For example, the report of systematic review and meta-analyses of RCTs reported that primary school nutrition interventions implemented in Asia are effective in decreasing BMI and BAZ among school-aged children (53). A healthy school food environment has the potential to play an important role in controlling a BMI since it could influence students' diet (54, 55) and diet is a key factor in determining and reducing overweight and obesity (56). These observations can justify the use of school in obesity prevention programs.

On the contrary, for a unit increase in family size the BAZ score will decrease by 0.06 (β 0.06, 95% CI −0.12–−0.01). Similarly, adolescents with a middle tertile wealth index were decrease BAZ score by 0.30 (β 0.30, 95% CI −0.55–−0.05) as compared to the higher tertile wealth index.

### Strength and limitation

The strength of this study was applying a 6-month prospective cohort study to show the cause and the effect relation of school feeding programs on BAZ score. Even though we have tried to control for potential confounders in our study there may be residual confounders from unmeasured variables. Furthermore, small sample size could be considered as a limitation.

## Conclusions

School feeding was positively associated with a change in BAZ score whereas family size and middle tertile wealth index were negatively associated. This implies that school feeding can serve as an optimal strategy for addressing the nutrition needs of adolescents.

### Recommendation

As human capital development of children is vital for a country in many ways, investing in it has proven to help children achieve their best as well as create productivity, stability, and improve resilience in communities. The Government of Ethiopia devotes significant funding to sustain the program.

These program require multisector involvement, strong institutional resource management, capacity building, and linkages with non-governmental fund-raising organizations (World Bank, WFP, FAO).

Finally, it is important to point out the need for a further RCT study which addresses the sustainability and potential long-term impacts of the program for a better policy implication.

## Data availability statement

The original contributions presented in the study are included in the article/supplementary material, further inquiries can be directed to the corresponding author.

## Ethics statement

The studies involving human participants were reviewed and approved by ethical approval and clearance were obtained from the Jimma University, Institute of Health, Institutional Review Board with a reference number of IRB/261/2020. Written informed consent to participate in this study was provided by the participants' legal guardian/next of kin.

## Author contributions

BM, KA, TB, and SK designed and supervised the study and ensured quality of the data and made a substantial contribution to the local implementation of the study and assisted in the analysis and interpretation of the data. BM did the analysis and drafted the manuscript and had the responsibility to submit the manuscript for publication. All authors critically reviewed the manuscript.
