# Corrigendum: Effect of school feeding program on body mass index of primary school adolescents in Addis Ababa, Ethiopia: a prospective cohort study

**DOI:** 10.3389/fnut.2023.1339283

**Published:** 2023-12-06

**Authors:** Bekri Mohammed, Tefera Belachew, Shemsu Kedir, Kalkidan Hassen Abate

**Affiliations:** ^1^Department of Human Nutrition, Institute of Public Health, College of Medicine and Health Sciences, University of Gondar, Gondar, Ethiopia; ^2^Department of Nutrition and Dietetics, Food and Nutrition Research Institute, Jimma University, Jimma, Ethiopia; ^3^Department of Public Health, College of Medicine and Health Science, Werabe University, Werabe, Ethiopia

**Keywords:** school feeding program, body mass index, adolescents, BAZ, school

In the published article, there was an error regarding the affiliation for Bekri Mohammed. As well as having affiliation [1], they should also have: ^2^Department of Nutrition and Dietetics, Food and Nutrition Research Institute, Jimma University, Jimma, Ethiopia.

The authors apologize for this error and state that this does not change the scientific conclusions of the article in any way. The original article has been updated.

